# Inhibition of microRNA-155 Reduces Neuropathic Pain During Chemotherapeutic Bortezomib via Engagement of Neuroinflammation

**DOI:** 10.3389/fonc.2020.00416

**Published:** 2020-03-31

**Authors:** Zongsheng Duan, Jian Zhang, Jing Li, Xiaochuan Pang, Hushan Wang

**Affiliations:** ^1^Department of Anesthesiology, The First Hospital of Jilin University, Changchun, China; ^2^Department of Radiology, The Second Part of The First Hospital of Jilin University, Changchun, China; ^3^Clinical Laboratory, The First Hospital of Jilin University, Changchun, China

**Keywords:** microRNA-155, bortezomib, chemotherapeutics, multiple myeloma, neuropathy

## Abstract

As a chemotherapeutic agent, bortezomib (BTZ) is used for the treatment of multiple myeloma with adverse effect of painful peripheral neuropathy. Our current study was to determine the inhibitory effects of blocking microRNA-155 (miR-155) signal on BTZ-induced neuropathic pain and the underlying mechanisms. We employed real time RT-PCR and western blot analysis to examine the miR-155 and expression of *pro*−*inflammatory* tumor necrosis factor-α receptor (TNFR1) in the dorsal horn of the spinal cord. Its downstream signals p38-MAPK and JNK and transient receptor potential ankyrin 1 (TRPA1) were also determined. Mechanical pain and cold sensitivity were assessed by behavioral test. In result, inhibition of miR-155 significantly attenuated mechanical allodynia and thermal hyperalgesia in BTZ rats, which was accompanied with decreasing expression of TNFR1, p38-MAPK, JNK, and TRPA1. In contrast, miRNA-155 mimics amplified TNFR1-TRPA1 pathway and augmented mechanical pain and cold sensitivity. In addition, mechanical and thermal hypersensitivity induced by miRNA-155 mimics were attenuated after blocking TNFR1, p38-MAPK, JNK, and TRPA1. Overall, we show the key role of miR-155 in modifying BTZ-induced neuropathic pain through TNFR1-TRPA1 pathway, suggesting that miR-155 is a potential target in preventing neuropathic pain development during intervention of BTZ.

## Introduction

Bortezomib (BTZ) is an inhibitor of the proteasome complex and is primarily used to treat multiple myeloma (1, 2). Nonetheless, one of its adverse effects is dose-limiting peripheral neuropathy (3). Because of a poor understanding of the mechanisms leading to BTZ-induced pain, treatment options have been limited. Therefore, it is noteworthy to determine molecular mediators of BTZ-induced neuropathy in order to provide a base for application of drugs and further to make therapeutic strategies with chemotherapeutic in patients with multiple myeloma.

MicroRNAs (miRNAs) are small noncoding endogenous RNA molecules, repressing their target mRNA through complementary binding in the message 3′-UTR (4). They have important effects in processes of multiple physiological responses including cell death and survival, cellular response to stress, stem cell division, and pluripotency (5). MiRNAs also contribute to disease processes such as cancer, cardiovascular disease and neurodegenerative diseases (6–8). As a result of their small size, relative ease of delivery, and sequence specificity in recognizing their targets, miRNAs are reflected as therapeutic targets of drug development (9). Notably, miRNA-155 (miR-155) plays a role in various physiological and pathological processes among various miRNAs (10–13). For instance, MiR-155 is involved in chronic immune activity through T cells by the downregulation of lymphocyte-associated antigens (14). In autoimmune diseases, miR-155 is found in patients' tissues and synovial fibroblasts (12). In multiple sclerosis, miR-155 is upregulated in resident myeloid cells of the nervous systems, blood monocytes and activated microglia (15).

The inflammatory process is involved in neuropathic pain (16, 17). Proinflammatory cytokines (PICs), including interleukin-1β (IL-1β), interleukin-6 (IL-6) and tumor necrosis factor-α (TNF-α), are elevated in the nervous system after nerve injury and/or inflammation, responsible for mechanical and thermal hypersensitization (18). In particular, TNF-α has a role in regulating neuropathic pain (16, 17). In pain models, TNF-α in sensory nerves is upregulated following peripheral nerve injury (19). TNF-α evokes hyperalgesia and allodynia in naive rats (20). Chemotherapeutic drugs paclitaxel or vincristine upregulate TNF-α (20). A blockade of TNF-α with its inhibitor or genetic impairment attenuates mechanical hyperalgesia and allodynia (19). BTZ treatment increases TNF-α in dorsal root ganglion (DRG) and spinal dorsal horn (21, 22) and TNF-α antibody inhibits allodynia by BTZ (23).

Transient receptor potential ankyrin 1 (TRPA1) has a functional role in regulating pain and neurogenic inflammation due to channel activation to various compounds (24–28). TRPA1 is presented in sensory nerves (27) and is involved in mechanical and cold hypersensitivity (29, 30). Further studies indicate that TRPA1 mediates mechanical and cold hypersensitivity by chemotherapeutics (31, 32). A recent study showed that a blockade of TNF-α signal attenuates intracellular p38-MAPK and JNK in the DRG and this alleviates mechanical hyperalgesia and cold hypersensitivity by BTZ via decreasing TRPA1 expression (33).

Nonetheless, it remains unrevealed for the role of miRNA-155 in control of TNF-α signal in neuropathy by BTZ. Accordingly, we examined the role played by miR-155 in modulating neuropathic pain after BTZ therapy. We hypothesized that inhibition of miR-155 alleviates mechanical hyperalgesia and cold hypersensitivity by BTZ. We also hypothesized that inhibition of miRNA-155 attenuates upregulation of TNF-α receptor (TNFR1), intracellular p38-MAPK and JNK signal and TRPA1 in the dorsal horn. We further hypothesized that blocking TNFR1-TRPA1 signal attenuates pain hypersensitivity induced by miRNA-155 mimics.

## Materials and Methods

### Animals

We performed all animal protocols in accordance with the guidelines of the International Association for the Study of Pain, approved by the Research Committee of our institution. Wistar rats (200–250 g) had free access to food and water and they were housed in individual cages in a temperature-controlled room on a 12/12 h light/dark cycle.

### Development of Neuropathic Pain and Intrathecal Administration

On the basis of our previous report (34), “BTZ (0.4 mg/kg body weight; dissolved in saline; Haoran BioTech Co., Shanghai, China) was given intraperitoneally (i.p.) once daily for five consecutive days. Control animals were given with an equivalent volume of vehicle (saline).”

Three days prior to each experiment, sodium pentobarbital (60 mg/kg, i.p.) was used to anesthetize the rats to implant intrathecal catheter for administration of drugs. In brief, one end of polyethylene-10 tubing was inserted intrathecally through an incision in the cisternal membrane and advanced 7–9 cm caudal until the tip of the catheter was positioned at the lumbar spinal level (L5 to L6). The other end of the intrathecal tubing was sutured to the musculature and skin at the incision site and externalized to the back of the rat.

**Figure 1:**
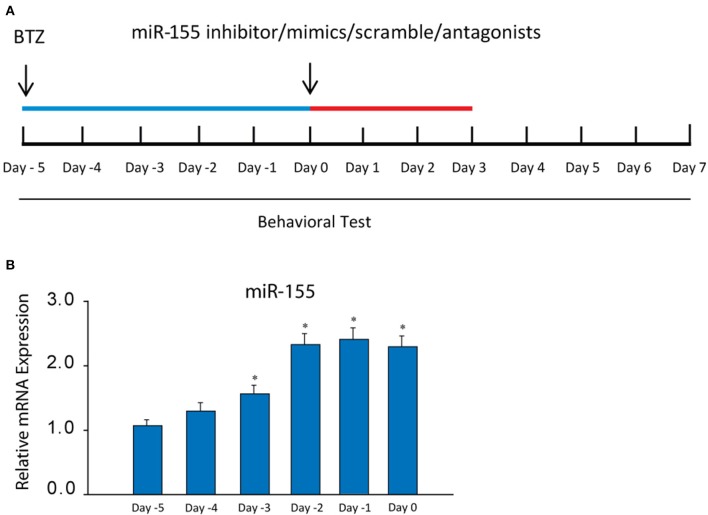
**(A)** A schematic diagram showing the schedule giving miR-155 inhibitor and mimics, their scrambles and receptor blockers as well as experimental protocols. BTZ was injected [intraperitoneally (i.p.), daily, 0.4 mg/kg body weight] over 5 days (indicated by blue bar). The starting day is expressed as “day −5.” An equivalent volume of vehicle was given in control animals. Then, miR-155 inhibitor/mimics, their scrambles, PTX, HC030031, SB203580, and SP600125 were administered (indicated as red bar) at the end of BTZ injection (day 0), which was also marked as day 0 in the Figures for results examining neuropathic pain. **(B)** showing that the levels of miR-155 mRNA were increased in the dorsal horn of the spinal cord after administration of BTZ and remained at a high level. *P < 0.05 vs. its level at days −5 and −4. The number of rats = 8–10 in each group.

After the end of BTZ administration, the following intrathecal injection was performed each day for three consecutive days: miR-155 inhibitor (sequence: 5′AAU UAC GAU UAG CAC UAU CCC CA-3′; 3 μg, Biomics Biotech, Nantong, China), miR-155 mimics (sequence: 5′-UUA AUG CUA AUC GUG AUA GGG GU-3′; 3 μg, Biomics Biotech, Nantong, China) and their corresponding scramble for negative controls (3 μg, Biomics Biotech, Nantong, China). MiR-155 inhibitor, miR-155 mimics and their respective scramble were dissolved in artificial cerebrospinal fluid (aCSF) before they were used; and their dosages used in this study were based on the published work (35). TNF-α synthesis inhibitor pentoxifylline (PTX, 10 μg), TRPA1 antagonist HC030031 (5 μg), p38-MAPK inhibitor SB203580 (5 μg), and JNK inhibitor SP600125 (5 μg) were also given by individual intrathecal injection each day for three consecutive days. Those drugs were obtained from Sigma Co. (St. Louis, MO, US) and all drugs were dissolved in aCSF before they were used. In each experiment, a Hamilton microsyringe (250 μL) was connected to the intrathecal tubing to make 100 μl of delivery. A schematic diagram of experimental protocols and the schedule giving drugs was shown in Figure 1A.

### Real-Time PCR

As described in our previous publication (35), “the tissues of the L5-L6 spinal cord dorsal horn were processed for the extraction of total RNA (RNeasy Mini Kit; Qiagen). RT-PCR was performed using the TaqmanW Universal PCR Master Mix and 18 s rRNA (TaqmanW PDAR) was used as an endogenous control to correct for variations in the samples. RT-PCR was performed in duplicate in 96-well plates containing 2 μL of cDNA. The thermal conditions of the cycles were 50°C/2 min, 60°C/30 min, and 95°C/5 min; and this was followed by 40 cycles at 94°C for 20 s and 62°C for 60 s. The ABI PRISM SDS 7000 thermal cycler was used to obtain the data. Using the 2-ΔΔCt comparative method, relative quantification of target gene was implemented and the threshold cycle value was defined by the point at which there was a statistically significant detectable increase in fluorescence.”

### Western Blot Analysis

As described in our previous publication (34), “the samples containing total protein of the L5-L6 dorsal horn tissues were extracted, centrifuged and the supernatants were collected to assess protein concentrations. Then the supernatant (containing 20 μg of protein) were loaded onto gels and electrically transferred to the membrane. The membrane was incubated overnight with primary rabbit antibodies (1:500), namely anti-TNFR1 (Abcam #ab90463), anti-TRPA1 (Novus Bio, NB100-91319), anti-p-p38-MAPK (USBio, USB#403230) and anti-p-JNK1 (Abcam #ab47337). The primary antibodies were purchased from Abcam Co and/or Antibodies-online Inc. After being washed, the membranes were incubated with anti-rabbit secondary antibody (1:1000, Sigma Co). The bands recognized by immunoreactive proteins were visualized by exposure of the membrane onto an x-ray film. The Scion Image software was employed to determine the optical density of immunoreactive proteins bands.”

### Behavioral Test

As described previously in our publications (34, 35), “we examined mechanical paw withdrawal threshold (PWT) of rat hindpaw in response to the stimulation of von Frey filaments. In brief, the filaments were bent for 5–10 s in our protocols. If a response was seen, we applied the filament of next lower force. If a response was not seen, we applied the filament of next greater force. To avoid injury during tests, the cutoff strength of the von Frey filament was 18 g. The tactile stimulus having a 50% likelihood of withdrawal was determined by the “up-down” method (36). We repeated each trial 2 times at ~2 min intervals. The mean value was used as the force producing a withdrawal response.”

We used Thermal Place Preference System to execute the thermal place preference test to assess a cold avoidance behavior as described in our previous study (34, 35). “We kept the first plate at neutral temperature (25°C) and the second plate at cold temperature (12°C). The test was executed in darkness and lasted 3 min and we left the rats free to explore both plates. The time spent on the cold plate was recorded using an infrared camera connected to a computer. To avoid learning or any place preference unrelated to cold, we inverted the temperature of the plates between two consecutive sessions. The animal studies were performed in a blind manner. Cold sensitivity was expressed as % time spent on the cold plate over 3 min [time on cold plate (seconds) /180 s × %].”

### Statistical Analysis

We used SPSS for Windows version 13.0 to perform all statistical analyses; and all data were analyzed using a two-way repeated-measures analysis of variance with Tukey's *post hoc* tests. We presented values as means ± standard error of mean. For all analyses of this study, differences were considered significant at *P* < 0.05.

## Results

### MiR-155 After BTZ Intervention

First, we determined the changes of miR-155 evoked by BTZ. An increase of miR-155 was seen 3 days after the beginning of BTZ intervention (Figure 1B). According to this result, in the rest of experiments the time point (day 0) was selected for injection miR-155 inhibitor and mimics, and receptor antagonists.

### Effects of MiR-155 TNFR1-TRPA1 Signal

**Figure 2:**
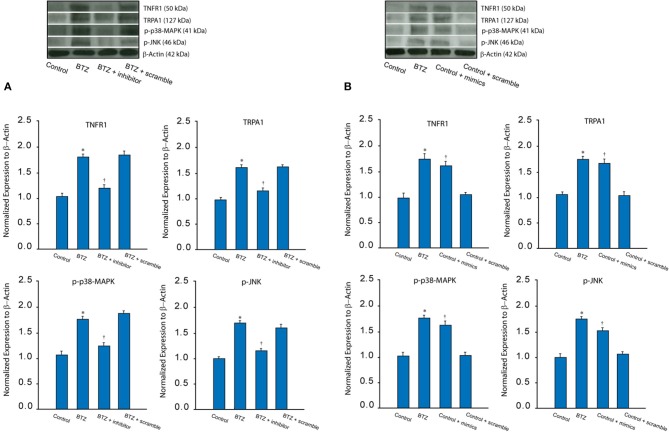
**(A)** Typical bands and averaged data show that BTZ increased the protein levels of TNFR1 (a subtype TNF-α receptor) and TRPA1 as well as intracellular signal p-p38-MAPK and p-JNK in the dorsal horn of the spinal cord as compared with controls. Furthermore, miR-155 inhibitor attenuated increases of these receptor and signal pathways in BTZ animals. Note that miR-155 inhibitor scramble did not alter upregulation of TNFR1, TRPA1 p-p38-MAPK/p-JNK induced by BTZ. *P < 0.05 vs. control rats and BTZ rats with inhibitor. †*P* < 0.05, BTZ rats with inhibitor vs. BTZ rats with scramble. *n* = 8–12 in each group. **(B)** Typical bands and averaged data show that TNFR1, TRPA1 and p-p38-MAPK/p-JNK were increased in the dorsal horn of control rats after intrathecal injection of miR-155 mimics; whereas those expressions were not altered after intrathecal injection of scramble. *P < 0.05 vs. control rats. †*P* < 0.05, control rats with mimics vs. control rats and control rats with scramble. *n* = 8–12 in each group.

In addition, our data presented in Figure 2A show that TNFR1, TRPA1 and p-p38-MAPK/p-JNK were increased in BTZ rats (*P* < 0.05, BTZ vs. control). Application of miR-155 inhibitor had attenuating effects on TNFR1-TRPA1 signal expression at the protein levels (*P* < 0.05, BTZ with inhibitor vs. BTZ and BTZ with scramble). In this experiment, protein expression of TNFR1, TRPA1 and p-p38-MAPK/p-JNK were not observed to be changed after application of miR-155 inhibitor scramble (*P* > 0.05, BTZ vs. BTZ with scramble).

Furthermore, we examined the effect of miR-155 mimics on TNFR1-TRPA1 signal pathways in control rats. Figure 2B shows that miR-155 mimics, but not its scramble, amplified TNFR1, TRPA1 and p-p38-MAPK/p-JNK (*P* < 0.05, control with mimics vs. control and control with scramble). Insignificant difference was seen in TNFR1-TRPA1 between control rats and control rats with miR-155 mimics scramble (*P* > 0.05).

### Effects of MiR-155 on Pain Response

**Figure 3:**
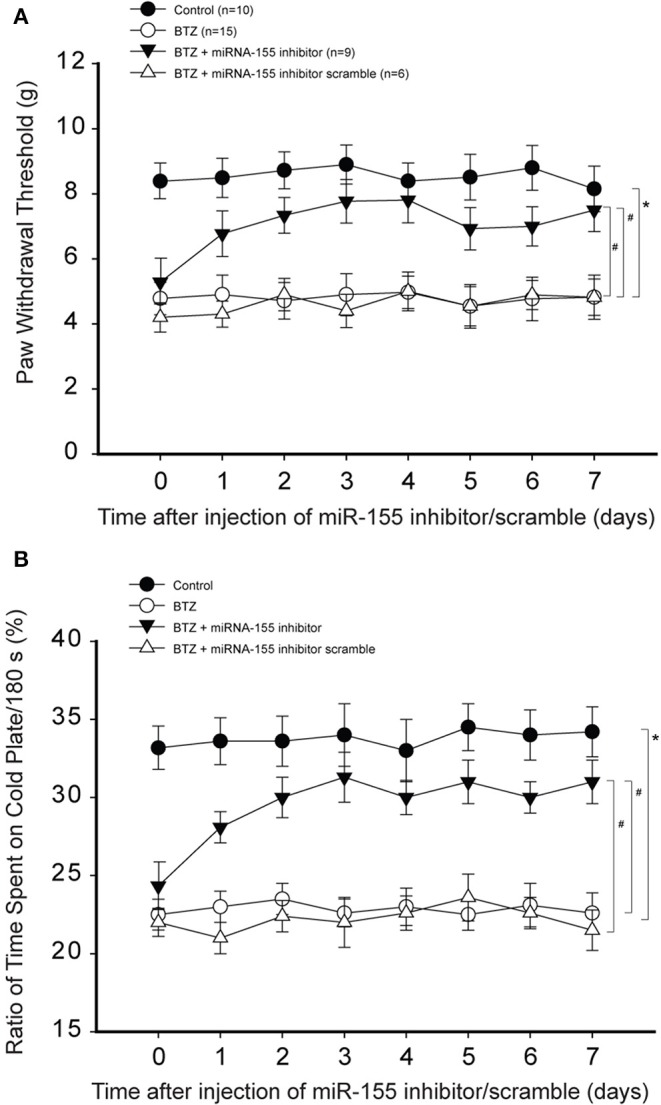
Effects of miR-155 inhibitor on mechanical and cold sensitivity. Paw withdrawal threshold (PWT) and cold sensitivity expressed as time spent on the cold plate (%) were examined in control rats and BTZ rats. **(A)** PWT was smaller in BTZ than in control rats at different time courses. Intrathecal injection of miR-155 inhibitor (3 μg each day over three consecutive days), but not its scramble, increased PWT in BTZ rats 1 day after its injection. **(B)** showing that time spent on the cold plate was less in BTZ rats than in control rats. MiR-155 inhibitor elevated % time spent on the cold plates in BTZ rats 1 day after its injection. Mir-155 inhibitor scramble had no effects on % time spent on the cold plates in BTZ rats. *P < 0.05, BTZ rats vs. control rats. #*P* < 0.05, BTZ rats with inhibitor vs. BTZ rats and BTZ rats with scramble. The number of rats in each group was shown on the figure.

Less PWT was seen in BTZ rats compared with control rats (Figure 3A). PWT was amplified 1 day after miR-155 inhibitor in BTZ rats and this effect was observed to last for 7 days (*P* < 0.05, BTZ vs. BTZ with inhibitor). In comparison with miR-155 inhibitor, its scramble failed to alter PWT in BTZ rats (*P* > 0.05, BTZ vs. BTZ with scramble). Figure 3B shows that BTZ decreased % time spent on the cold plate. MiR-155 inhibitor increased % time spent on the cold plate in BTZ rats (*P* < 0.05, BTZ vs. BTZ with inhibitor). Similarly, miR-155 inhibitor scramble had no effects in BTZ rats (*P* > 0.05, BTZ vs. BTZ with scramble).

**Figure 4:**
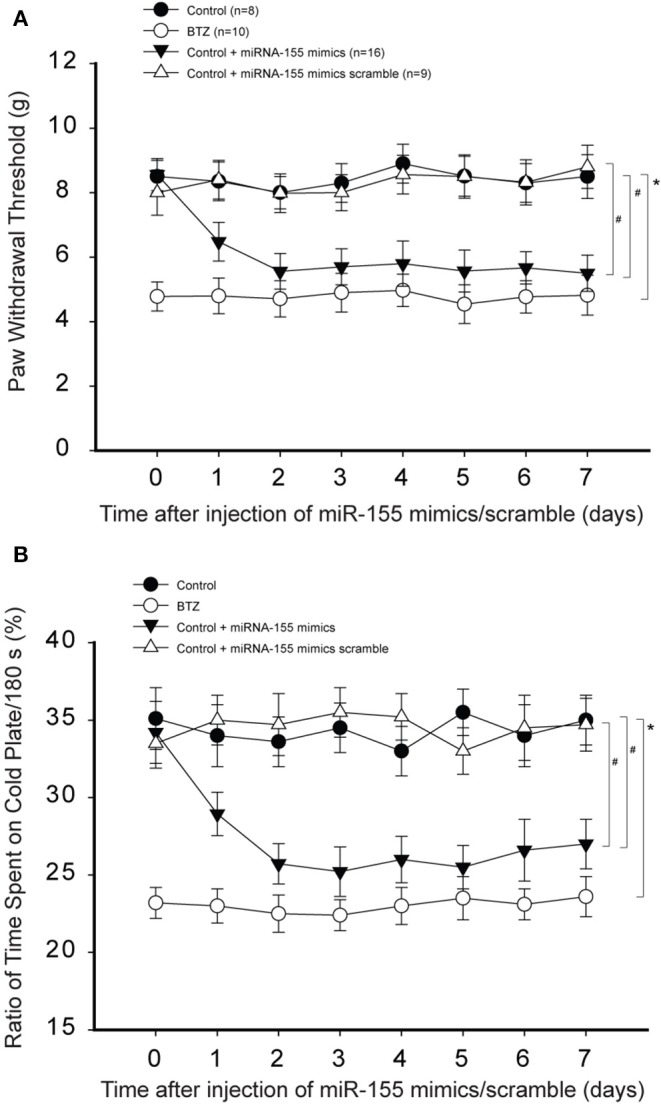
Effects of miR-155 mimics on mechanical and cold sensitivity. Paw withdrawal threshold (PWT) and cold sensitivity expressed as time spent on the cold plate (%) were examined in control rats and BTZ rats. **(A)** Intrathecal injection of miR-155 mimics (3 μg each day over three consecutive days) decreased PWT in control rats 1 day after its injection as compared with scramble. **(B)** showing that time spent on the cold plate was less in BTZ rats than in control rats. In control rats, miR-155 mimics decreased % time spent on the cold plates one day after its injection, but miR-155 mimics scramble did not change % time spent on the cold plates. *P < 0.05, BTZ rats vs. control rats. #*P* < 0.05, control rats with mimics vs. control rats and control rats with scramble. The number of rats in each group was shown on the figure.

Next, we determined the effects of miR-155 mimics on PWT and % time spent on the cold plate in control rats. Figure 4A demonstrates that miR-155 mimics decreased PWT in control rats (*P* < 0.05, control vs. control with mimics), but miR-155 mimics scramble did not have significant effects on PWT. Similarly, Figure 4B shows that miR-155 mimics, but not its scramble, decreased % time spent on the cold plate in control rats (*P* < 0.05, control with mimics vs. control and control with scramble).

### Effects of Inhibiting TNFR1-TRPA1 Pathway

**Figure 5:**
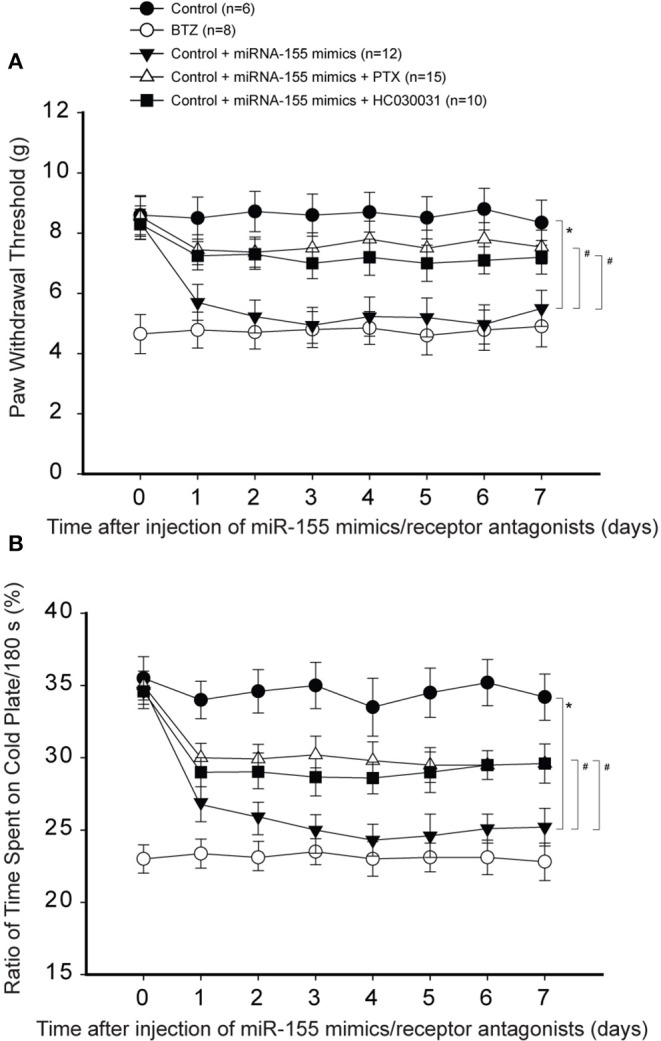
Effects of blocking TNFR1 and TRPA1 on mechanical and cold sensitivity. TNF-α signal was inhibited by pentoxifylline (PTX; 10 μg each day over three consecutive days). TRPA1 was blocked by administration of HC030031 (5 μg each day over three consecutive days). **(A)** PWT was smaller in BTZ than that in control rats for all time courses. As compared with controls, intrathecal injection of miR-155 mimics (3 μg each day over three consecutive days) significantly decreased PWT, and the effects of miR-155 mimics were attenuated by injection of PTX and HC030031. **(B)** showing that % time spent on the cold plate was less in BTZ rats and in rats with injection of miR-155 mimics. PTX and HC030031 attenuated the effects of miR-155 mimics. *P < 0.05, control rats vs. control rats with mimics; #*P* < 0.05, rats with mimics vs. rats with mimics plus PTX or mimics plus HC030031. The number of rats in each group was shown on the figure.

We were to determine if the effects of miR-155 were via TNFR1-TRPA1 signals. Thus, we examined if blocking those signal pathways can attenuate hypersensitivity of mechanical and cold stimulation induced by miR-155 mimics in control rats. Figures 5A,B show that miR-155 mimics decreased PWT and decreased % time spent on the cold plate in control rats. As PTX or HC030031 was given by intrathecal injection, decreases in PWT and % time spent on the cold plate were mostly improved in rats with miR-155 mimics (*P* < 0.05, rats with mimics vs. rats with mimics plus PTX or HC030031).

**Figure 6:**
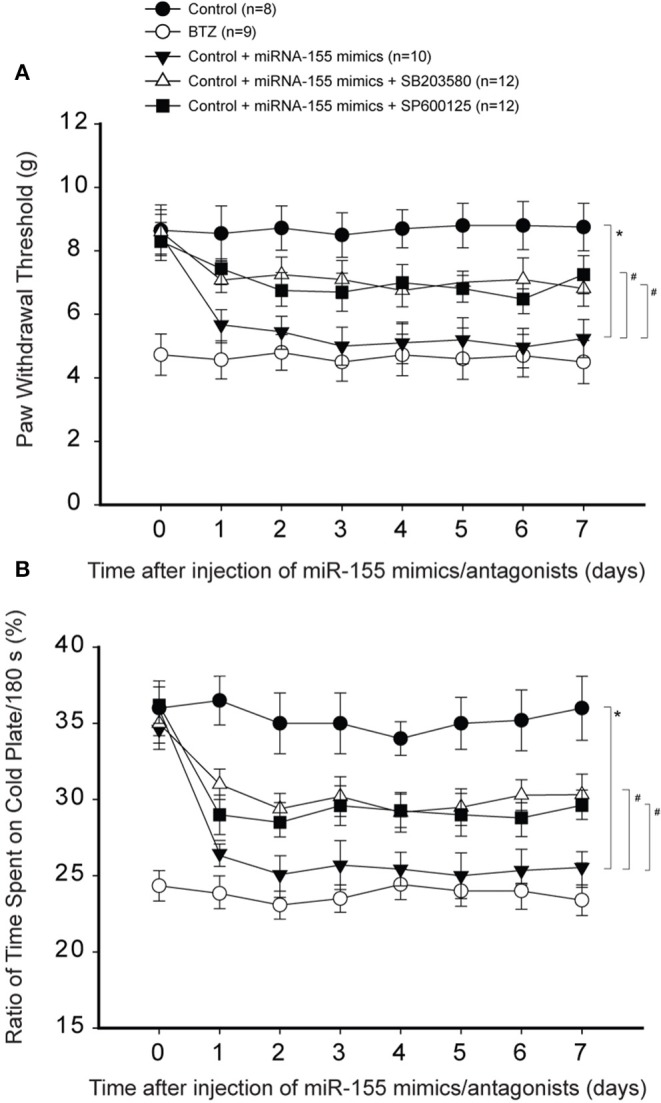
Effects of blocking p38-MAPK and JNK signal on mechanical and cold sensitivity. Intrathecal injection of SB203580 (5 μg each day over three consecutive days) and SP600125 (5 μg each day over three consecutive days) were performed to inhibit p38-MAPK and JNK signal pathway. **(A,B)** As compared with controls, intrathecal injection of miR-155 mimics (3 μg each day over three consecutive days) significantly decreased PWT and % time spent on the cold plate. With respective SB203580 and SP600125, decreased PWT and % time spent on the cold plate by miR-155 mimics were largely restored. *P < 0.05, control rats vs. control rats with mimics; #*P* < 0.05, rats with mimics vs. rats with mimics plus SB203580 or mimics plus SP600125. The number of rats in each group was shown on the figure.

Likewise, Figures 6A,B demonstrate that inhibiting p38-MAPK and JNK using respective SB203580 and SP600125 also attenuated reductions in PWT and % time spent on the cold plate in animals with miR-155 mimics (*P* < 0.05, rats with mimics vs. rats with mimics plus SB203580 or SP600125).

## Discussion

With progression of cancer, pain is one of the most common and distressing symptoms suffered by patients (37). Cancer pain can be due to a tumor compressing; nerve and other tissue changes caused by a hormone and immune responses; and/or treatments and diagnostic procedures (37, 38). Notably, pain caused by chemotherapy can continue even after the end of treatment (37, 39, 40). Therefore, cancer pain during chemotherapy is considered a significant issue in clinics.

BTZ is frequently applied for treatment of multiple myeloma (1–3), but painful neuropathy and heightened cold sensitivity are main complications during application of BTZ (3). Of note, BTZ induces neuropathic pain in rats after initiation of its chemotherapy (21–23). Indeed, the abnormal sensation was ablated many days after BTZ (21–23). Using this rat model, a recent study showed that blocking TNF-α and TRPA1 signal in the DRG attenuated mechanical hyperalgesia and cold hypersensitivity following BTZ, and the effects were via engagement of intracellular signals p38-MAPK and p-JNK (33).

The sensory nerves (neurons) and dorsal horn of the spinal cord paly a primary role in conducting signals of pain responses (16, 17). The prior reports suggest that TNF-α is increased in DRG and spinal dorsal horn after administration of BTZ (21, 22). We also observed that BTZ increased TNFR1-TRPA1 in the dorsal horn of rats. Interestingly, intrathecal application of miR-155 inhibitor attenuated TNFR1-TRPA1 and p38-MAPK and p-JNK in the dorsal horn of BTZ rats. This also attenuated mechanical hyperalgesia and cold hypersensitivity by BTZ. In contrast, miR-155 mimics injected into the dorsal horn of control rats amplified TNFR1-TRPA1 and led to mechanical hyperalgesia and cold hypersensitivity. In addition, blocking TNFR1-TRPA1 and p38-MAPK/p-JNK attenuated amplified pain by intrathecal injection of miR-155 mimics. Furthermore, we showed that miR-155 inhibitor decreased the protein levels of TRPA1 in BTZ rats. Likewise, miR-155 mimics increased the protein levels of TRPA1 in control rats. These results suggest that there is an interaction between miR-155 signal and activation of TRPA1 pathway. Overall, our data suggest the role of miR-155 in modifying mechanical hyperalgesia and cold hypersensitivity during BTZ therapy.

In general, miR-155 is involved with disease processes related to inflammation. For instance, deficiency of miR-155 inhibits IL-17 and decreases renal damage in nephropathy (41). The lack of miR-155 can decrease production of B and T cells in autoimmune arthritis (42). A high level of miR-155 is observed in patients with gouty arthritis compared with healthy controls (43). It was also reported that deficiency in miR-155 alleviates inflammatory bowel disease through downregulation of the Th1/Th17 (44). We demonstrated that BTZ amplifies miR-155 in the dorsal horn and miR-155 inhibitor can attenuate BTZ-induced TNF-α signal pathway leading to decreases of neuropathic pain. Moreover, miR-155 mimics amplifies pain response to mechanical and thermal stimulation.

Anti-inflammatory protein suppressor of cytokine signaling 1 (SOCS1) is a target gene of miR-155 (45). SOCS1 is a key regulator of inflammatory signals, negatively regulating the inflammation feedback (46). Deficiency of SOCS1 leads to amplified responsiveness to inflammatory stimuli in various cells or in animals (47, 48). In a prior study, the role of miR-155 and SOCS1 in neuropathic pain was identified (49). This prior study showed that miR-155 was upregulated in the dorsal horn after chronic constriction injury. MiR-155 inhibitor alleviated neuroinflammation and neuropathic pain. Also, miR-155 inhibitor suppressed NF-κB and p38-MAPK activation via SOCS1. Consistent with this previous finding, our current results showed that miR-155 mRNA is elevated in the dorsal horn after administration of BTZ and this increase remains for several days. This is accompanied with upregulation of protein expression of p38-MAPK as well as JNK. MiR-155 inhibitor attenuates p38-MAPK signal and attenuates BTZ-induced mechanical and thermal hypersensitivity. Thus, miR-155 is likely a potential target for the therapeutic intervention of neuropathic pain during BTZ therapy.

A variety of miRNAs has the role in pain processing in experimental and clinical pain (50). For instance, miR-203 is involved in neuropathic pain through Rap1a and its downstream signal MEK/ERK (51). Inhibition of miR-21 attenuates neuropathic pain in rats (52). Activation of miR-195 by peripheral nerve injury aggravates neuropathic pain via autophagy and this also leads to increases of IL-1β, and TNF-α (53). Thus, further studies are needed to determine the networks of miRNAs in engagement of neuropathic pain.

In general, cytotoxic drugs used for chemotherapy activate oxidative stress signals and thereby induce peripheral neuropathy (35, 54, 55). The previous study has demonstrated that TRPA1 is activated in the process of BTZ-induced pain and oxidative stress is involved in activation of the signal pathways (55). In this previous study (55), BTZ evoked mechanical, cold, and selective chemical hypersensitivity in mice and the effects of BTZ are reverted by treatment with the TRPA1 antagonist HC-030031 and by the oxidative stress scavenger α-lipoic acid. Thus, it is speculated that activation of oxidative stress signal is likely involved in the role of miR-155 in BTZ-induced neuropathic pain in our current study.

Neuropathic pain can last ~30 days after the end of BTZ administration. We need to acknowledge a study limitation of the current report that we did not examine pain response >7 days after BTZ in this report. We examined the effects of miR-155 inhibitor and miR-155 mimics on neuropathic pain 1–7 days after BTZ. Our design was based on the reasons: (1) significant pain was developed after a few days after BTZ; and (2) we performed intrathecal delivery of inhibitor/mimics for three consecutive days. The main focus of this study was to determine if miR-155 inhibitor can attenuate neuropathic pain induced by BTZ. If we can show the effectiveness of inhibitor 7 days after BTZ, this would indicate that its effects can last longer with a longer usage of inhibitor (i.e., 30 days after BTZ). Nonetheless, we have shown that miR-155 inhibitor alleviated pain response 7 days after BTZ in our current report.

In conclusion, the protein expression levels of TNFR1 and TRPA1 and intracellular p38-MAPK/JNK in the dorsal horn are upregulated by BTZ. Inhibition of miR-155 decreased those signal pathway expression and attenuated mechanical allodynia and thermal hyperalgesia in BTZ rats. In addition, miRNA-155 mimics increased mechanical pain and cold sensitivity without injection of BTZ. Mechanical and thermal hypersensitivity induced by miRNA-155 mimics were attenuated after blocking TNFR1, TRPA, p38-MAPK, and JNK. We provided evidence for the role of miR-155 in regulating BTZ-induced neuropathic pain through TNFR1-TRPA1 pathway, having clinical implications that miR-155 is a potential target in preventing neuropathic pain during BTZ chemotherapeutics.

## Data Availability Statement

The datasets generated for this study are available on request to the corresponding author.

## Ethics Statement

The animal study was reviewed and approved by Institutional Animal Care and Use Committee of Jilin University.

## Author Contributions

ZD and JZ contributed to the data collection and analysis and drafted the paper. JL and XP contributed to the data analysis. HW contributed to the experimental designs of this study and oversaw all data collection and analysis and reviewed manuscript.

### Conflict of Interest

The authors declare that the research was conducted in the absence of any commercial or financial relationships that could be construed as a potential conflict of interest.
